# Role of Pattern Recognition Receptors in KSHV Infection

**DOI:** 10.3390/cancers10030085

**Published:** 2018-03-20

**Authors:** Timsy Uppal, Roni Sarkar, Ranjit Dhelaria, Subhash C. Verma

**Affiliations:** 1 Department of Microbiology and Immunology, University of Nevada, Reno, School of Medicine, 1664 N, Virginia Street, MS 320, Reno, NV 89557, USA; tuppal@medicine.nevada.edu (T.U.); rsarkar@medicine.nevada.edu (R.S.); 2 Department of Internal Medicine, UTMB Galveston, League City Campus, 301 University Blvd, Galveston, TX 77555, USA; rdhelaria@gmail.com

**Keywords:** innate immune responses, pattern recognition receptors, KSHV, interferons, cytokines, signaling pathways

## Abstract

Kaposi’s sarcoma-associated herpesvirus or Human herpesvirus-8 (KSHV/HHV-8), an oncogenic human herpesvirus and the leading cause of cancer in HIV-infected individuals, is a major public health concern with recurring reports of epidemics on a global level. The early detection of KSHV virus and subsequent activation of the antiviral immune response by the host’s immune system are crucial to prevent KSHV infection. The host’s immune system is an evolutionary conserved system that provides the most important line of defense against invading microbial pathogens, including viruses. Viruses are initially detected by the cells of the host innate immune system, which evoke concerted antiviral responses via the secretion of interferons (IFNs) and inflammatory cytokines/chemokines for elimination of the invaders. Type I IFN and cytokine gene expression are regulated by multiple intracellular signaling pathways that are activated by germline-encoded host sensors, i.e., pattern recognition receptors (PRRs) that recognize a conserved set of ligands, known as ‘pathogen-associated molecular patterns (PAMPs)’. On the contrary, persistent and dysregulated signaling of PRRs promotes numerous tumor-causing inflammatory events in various human cancers. Being an integral component of the mammalian innate immune response and due to their constitutive activation in tumor cells, targeting PRRs appears to be an effective strategy for tumor prevention and/or treatment. Cellular PRRs are known to respond to KSHV infection, and KSHV has been shown to be armed with an array of strategies to selectively inhibit cellular PRR-based immune sensing to its benefit. In particular, KSHV has acquired specific immunomodulatory genes to effectively subvert PRR responses during the early stages of primary infection, lytic reactivation and latency, for a successful establishment of a life-long persistent infection. The current review aims to comprehensively summarize the latest advances in our knowledge of role of PRRs in KSHV infections.

## 1. Introduction

Pathogens such as bacteria, fungi, viruses, and parasites (protozoa and worms) have long been implicated in several human malignancies, with viruses alone being identified as etiological agents of about 15–25% of different human cancers, worldwide [1,2,3]. Viruses, in general, are metabolically inert but can grow rapidly inside the host cell and kill the infected cell releasing progeny virions. Some viruses, such as tumor causing viruses or oncoviruses, on the other hand, may mediate transformation of an infected host cell into a cancer cell and may establish long-term persistent infection while maintaining low levels of viral replication. These oncoviruses either integrate into the host genome or persist as an episome and express viral oncogenes to instigate abnormal cellular proliferation and multistep tumor development (tumorigenesis). Tumor virus infections are generally asymptomatic however, it is the persistent nature of the latent and the lytic infection that triggers their oncogenic potential and stimulates tumor formation in their hosts. Presently, seven tumor-causing DNA/RNA viruses are known to exist and cause cancers in humans. They include Hepatitis B and C Viruses (HBV and HCV), Human Papillomavirus (HPV, Type 16 and 18), gammaherpesviruses namely, Epstein-Barr Virus (EBV) and Kaposi’s Sarcoma-associated Herpesvirus (KSHV), recently identified, Merkel Cell Polyomavirus (MCPyV) and Human T-cell lymphotrophic virus-1 (HTLV-1) [4,5]. Tumorigenesis is a multistep process, and although these seven oncogenic viruses follow diverse strategies and cause different human cancers, yet, as proposed by Weinburg et al. tumors in humans display some key biological characteristics, including the evasion of growth suppressors, apoptosis, induction of angiogenesis, invasion, metastasis, tumor-promoting inflammation, and evasion of immune destruction, to name a few [6,7]. These characteristics, often called “hallmarks of cancer”, are acquired by mammalian cells to transform, survive, proliferate and become tumorigenic. 

Decades of intensive tumor virus and associated cancer research have made it increasingly clear that the interplay between host-intrinsic immunity and virus-mediated tumorigenesis is complex. The host immune system is a surveillance system that, via numerous innate immune and adaptive immune responses, protects the host against the intruding pathogens, tissue damage and metabolic disorders [8,9]. The main components of the innate immune system (i.e., macrophages, dendritic cells, and natural killer cells) play a pivotal role in modulating viral infection and determining disease severity and outcome by triggering (i) activation of type I interferon (IFN-α/β) signaling, (ii) activation of multiple cellular cytokines and chemokines, and subsequent (iii) initiation of host adaptive (antigen-specific) immune signals [10]. Hence, it is quintessential for invading viruses to efficiently antagonize cellular immune responses and immune checkpoints, to secure viral infection. 

A growing body of information from clinical and epidemiological studies, along with studies using animal models of human cancer, provides evolving insights into the immune system as an important barrier to tumor formation and progression [11]. Research has also shed light on the involvement of immune suppression, and the role of immunosuppressive lymphoid and myeloid cells in the development of virus-induced tumors. For instance, a spontaneous tumor formation occurs in mice genetically deficient in IFN-γ, as compared to wild-type mice [12,13]. In addition, immune cell-deficient mice demonstrated increased susceptibility towards cell proliferation and tumor formation [11]. Further, severe immunosuppressed patients and individuals are reported to display higher incidence for viral oncogenicity [14,15,16]. KSHV/HHV-8-linked Kaposi’s Sarcoma (KS) and EBV-positive B-cell lymphomas are the most prominent tumors under these perturbed immune conditions [17,18,19]. 

## 2. KSHV Infections, Diagnosis and Treatment

### 2.1. KSHV Genome and Associated Malignancies

KSHV/HHV-8 is an oncogenic, large, enveloped, double-stranded (ds) DNA virus, that has been classified as class I carcinogen by International Agency for Research on Cancer (IARC). KSHV has been shown to effectively infect and persist in mammalian B-lymphocytes/endothelial cells (of different lineage) and can induce multiple sarcomas and lymphomas in the infected hosts. KSHV infection is unequivocally linked to KS, a leading cancer among untreated HIV-positive individuals or immune compromised organ transplant recipients [20,21]. The discovery of KSHV in 1994 from KS lesions played an instrumental role for our understanding of this disease [22]. KS is an unusual, aggressive, low-grade mesenchymal tumor of highly proliferative endothelial cells, characterized by infiltrative red/brown/purple lesions on the skin, mucosal surfaces, lymph nodes, and internal organs (such as lungs and digestive tracts) [21,23]. KS has been classified into four distinct epidemiological-clinical forms, i.e., classic/sporadic KS, a relatively aggressive endemic/African KS, an iatrogenic KS/post-transplant KS, and acquired immune deficiency syndrome (AIDS)-related epidemic KS [24,25,26,27]. Also, KSHV latent viral genome and gene products have been detected in all these forms of KS. KSHV has been characterized as a lymphotropic oncogenic herpesvirus, due to its association with two distinct B-cell lymphoproliferative diseases, namely, primary effusion lymphoma [20], and multicentric Castleman’s disease (MCD)-linked plasmablastic lymphoma [28]. PEL, also referred to as body cavity-based lymphoma (BCBL), is a B-cell non-Hodgkin’s lymphoma predominantly found in AIDS patients with compromised immune status, and is characterized by lymphomatous effusion tumor growth in body cavities. KSHV-MCD, on the other hand, is a rare but rapidly progressing polyclonal B-cell lymphoproliferative disorder which is characterized by vascular endothelial proliferation of the lymph nodes and lymphoid tissues in the KSHV/HIV-infected patients [29,30]. 

The infection with KSHV begins with the attachment of viral envelope glycoproteins to several host membrane receptors that are present on the cell surface of target cells [31]. Upon entry, the viral capsid is released in the cytoplasm and transported to the nuclear periphery followed by release of the viral genome into the nucleus [31,32,33]. Inside the nucleus, KSHV linear dsDNA is circularized and chromatinized to persist as a latent “minichromosome” or “episome”, which transcribes and replicates along with the host chromosome in proliferating infected cells for efficient long-term infection [31,32,33]. KSHV can exhibit either a latent, non-productive or a lytic, productive infection. However, like other gammaherpesviruses, KSHV prefers a prolonged latency phase with a restricted latent transcriptional program. The stringent default latency period is reversible, and changes in the host cell environment can reactivate the silent virus towards the lytic cycle, wherein tightly controlled and coordinated expression of multiple viral lytic genes allow viral genome amplification, virion assembly, and progeny virus production [34]. KSHV also displays a transient but abortive lytic cycle gene program following de novo infection, as some lytic transcripts have been detected in transcriptome analyses of KSHV-infected cells [35,36]. Indeed, KSHV can support different transcription profiles in lymphatic endothelial cells and blood vascular endothelial cells [37]. These findings imply that the lytic phase also plays a central role in the stable maintenance of the viral genome, an essential pre-requisite for the persistent infection and development of KSHV-induced tumors [38]. 

### 2.2. KS Diagnostic Markers and Therapeutic Approaches

**Figure 1:**
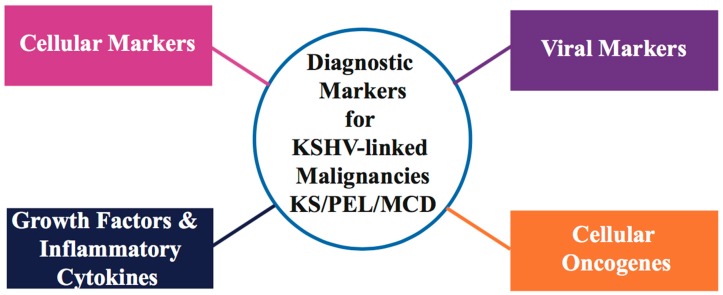
Summary of significant cancer diagnosis markers detected in Kaposi’s sarcoma-associated herpesvirus (KSHV)-associated neoplasms. These biomarkers can be categorized into virus specific markers (KSHV genome or specific proteins), cellular markers, cellular oncogenes, and inflammatory cytokines/growth factors.

Differential diagnosis of malignant, soft-tissue KS vascular tumors has been difficult, especially in the presence of other benign/malignant/non-vascular soft-tissue neoplasms. Immuno-histochemical staining and PCR detection of KSHV viral genome/DNA sequences in KS tissue are two major tools for diagnosis and clinical evaluation of KSHV-associated malignancies. Histologically, KS tumors are characterized by the presence of KSHV latency-associated nuclear antigen (LANA) [39] positive elongated spindle-like, poorly differentiated endothelial cells, and inflammatory infiltrates involving monocytes, B and T cells [40]. Studies have demonstrated that low peripheral blood CD4 lymphocyte count is a significant marker associated with AIDS-related KS [41]. As KS lesions harbor KSHV in its latent form, expression of KSHV latency transcripts, including major nuclear latency protein LANA (OR73), vFLIP (ORF71 or K13), vCyclin (ORF72), Kaposins (K12), and a cluster of viral microRNAs (miRNAs), serve as useful biomarkers of KS tumorigenesis [42,43,44,45]. LANA has been shown to dysregulate the p53 and pRb tumor suppressors in KSHV-infected cells [46,47]. Since KSHV can display both latent/lytic gene activity during KS infection, some KSHV lytic gene transcripts, such as vGPCR (viral G-protein coupled receptor), vIL-6 (viral Interleukin-6), vBcl2 homolog, vMIPs (viral macrophage inflammatory protein), and vIRF-1 and -3 (viral IFN regulatory factor), have also been detected in KS and PEL-tumor isolates [48,49,50]. Overexpression of vascular endothelial cell markers including CD36, CD138/syndecan-1 and factor XIII has been found in several KSHV latency models (Reviewed in [51,52,53]). Studies of KS tumors have found over-expression of selective lymphatic and blood vessel endothelial markers, such as LYVE-1 (Lymphatic vessel endothelial receptor-1), VEGFR-3 (Vascular endothelial growth factor receptor 3 precursor), PDPN (podoplanin), CXCR4 (Chemokine C-X-C motif receptor 4), DLL4 (Delta like protein 4), and CXCL12/SDF1 (Stromal cell derived factor 1) [54,55,56]. Rosado and colleagues have demonstrated the use of monoclonal antibodies directed against CD31, CD34, D2-40, and FLI1 (Friend leukemia integration 1 transcription factor) as specific and selective markers of endothelial differentiation in different clinical subtypes and tumor stages of KS [57]. Based on current available data, higher levels of inflammatory cytokines and pro-angiogenic growth factors, such as IFN-γ, interleukin 1/6 (IL-1/6), oncostatin M, TNF-α/β (tumor necrosis factor-α/β) and bFGFs (basic fibroblast growth factor) that favor the development of tumors, have been reported in HIV-positive KSHV-infected patients [58,59,60]. Detailed information on current biomarkers in KSHV-linked malignancies is summarized in Figure 1 and Table 1. 

Survival of patients with KS has remained poor due to a combination of poor prognosis and lack of effective treatment therapies. The current therapeutic approaches available for the treatment of KS patients include combined antiretroviral therapy (cART), cytotoxic chemotherapy (i.e., liposomal polyethylene glycol doxorubicin-Doxil), radiotherapy, and surgery [102,103]. Though cART improves KS, cART-initiated regression of KS has been linked to immune reconstitution inflammatory syndrome (IRIS) of varying severity in some HIV-infected patients [104]. Standard cytotoxic chemotherapy is effective; however, access to this treatment of choice is limited and rarely leads to complete responses in advanced KS [105]. Though a number of nucleoside analog-based antiviral drugs, such as ganciclovir, acyclovir, foscarnet, and cidofovir have been reported to induce KS regression, these drugs have significant toxicities and fewer case reports suggest reduced KS incidence [106,107,108,109]. Several novel drugs including cepharanthine, and diethyldithiocarbamate (NF-κB inhibitors), bevacizumab (angiogenesis inhibitors), sorafenib and imatinib (receptor tyrosine kinase inhibitors), and rapamycin (mTOR inhibitors), are currently being developed for the treatment of KSHV infection; further investigations are however required to determine their efficacy and tolerability [110,111,112,113,114]. Although several therapeutic approaches are showing promising advancement in the treatment of KSHV-linked disorders in the infected patients, unfortunately, there is no definite cure for KSHV infections. Hence, there is a growing need to devise newer virus-targeted therapies without the toxicity of chemotherapy for the prevention/early treatment of KSHV-positive malignancies. In this regard, a detailed understanding of interplay between KSHV and the infected host’s immune system could assist in the development of KSHV therapeutics. 

## 3. KSHV Inhibition of PRR-Dependent Immune Responses

**Figure 2:**
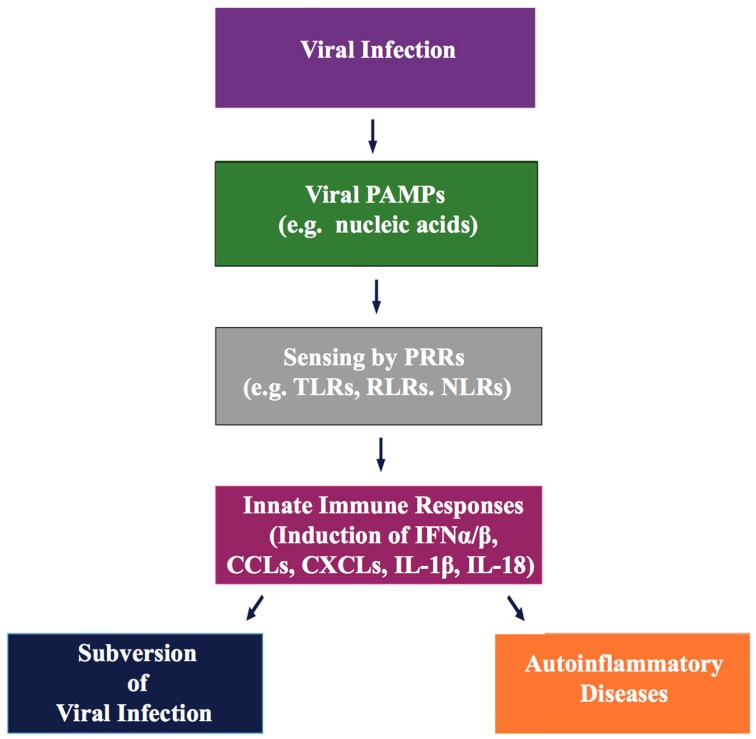
Schematic representation of the key principles involved in innate immune recognition by host pattern recognition receptors (PRRs). Upon viral infection, viral pathogen-associated molecular patterns (PAMPs), such as the viral genome or gene products are recognized by several cellular PRRs present on the cell membrane, in the cytoplasm or within intracellular compartments. Sensing of PAMPs by PRR triggers PRR-mediated signaling pathways and activates antiviral responses, leading to subversion of the infection. Abnormal activation of PRRs may also trigger auto-immunity. Abbreviations: TLRs, Toll-like receptors; NLRs, nucleotide oligomerization domain (NOD)-like receptors; RLRs, retinoic acid-inducible gene I (RIG-I)-like receptors; IFN-α/β, type-I interferons α/β; CCLs/CXCLs, chemokine ligands; IL-1β/IL-18, inflammatory cytokines.

Innate immunity is the first and most rapid line of host surveillance against intruding microbial pathogens. Host cell mediated intracellular discrimination of harmless “self” (cellular DNA/RNA) and harmful “non-self” (viral DNA/RNA) is mediated through germline-encoded proteins, known as pattern recognition receptors (PRRs). PRRs are located either on the cell surface, or within distinct cellular compartments of the cytosol, and respond to microbial molecular marks, known as pathogen-associated molecular patterns (PAMPs) (Figure 2) [115,116,117]. PAMPs are essential functional components of pathogens, such as microbial nucleic acids (dsDNA/dsRNA/single stranded (ss)RNA/5′-triphosphate RNA), lipopolysaccharide (LPS), lipoproteins, envelope glycoproteins, and peptidoglycans [117]. Sensing viral PAMPs, present in the incoming virus or released during viral replication, activate innate immune signals by cellular PRRs to produce type I interferon (IFNs) and multiple antiviral cytokines, that result in the robust expression of several antiviral proteins to promote elimination of invading viruses. Transient yet robust antiviral inflammatory cytokine activation not only recruits immune cells to the site of viral infection, but also stimulates antigen-specific adaptive immune responses to control the spread of the virus.

There are four major families of PRRs including Toll-like receptors (TLRs), nucleotide oligomerization domain (NOD)-like receptors (NLRs), retinoic acid-inducible gene I (RIG-I)-like receptors (RLRs) and intracellular DNA sensors (e.g., cGAS, IFI16) (Reviewed in [117,118,119]) that recognize virus-associated nucleic acids in the endosomes, nucleus, and cytoplasm of the infected cells and initiate multiple immunomodulatory and inflammatory pathways through a complex yet well-coordinated signaling system. The TLR family of receptors are membrane-bound receptors whereas RLRs and NLRs are cytosolic receptors [115]. During herpesvirus infection, the viral genome and viral latency/lytic transcripts (ssRNA/dsRNA) often serve as immunogenic molecular ligands to cellular PRRs (Reviewed in [114]).

### 3.1. TLRs in KSHV Infection

Members of the TLR family are the most extensively studied and characterized PRRs (Reviewed in [120]). TLRs are type-I transmembrane glycoproteins, and are expressed by macrophages, neutrophils, Dendritic Cells, Natural Killer cells, epithelial and endothelial cells. The mammalian TLR family comprises of 10 members, designated as TLRs 1–10. In comparison to humans, 12 TLRs have been identified in mice numbered as TLR 1–9 and TLR 11–13 (TLR 10 is identified as a pseudogene) [121]. All TLRs shares a common structural organization and consist of an ectodomain containing leucine-rich repeats (LRRs), a transmembrane domain and a cytoplasmic Toll/Interleukin-1 (IL-1) receptor signaling domain [122]. Cell-surface expressed TLRs (TLR-1, -2, -4, -5, -6 and -10) mainly recognize pathogenic wall components, including lipids, proteins and polysaccharides, whereas endosomal TLRs (TLR-3, -7, -8, and -9) sense microbial nuclei acids, such as, viral dsDNA and ssRNA [123]. In addition, TLRs can also sense endogenous danger-associated molecular patterns, such as host alarmins and inflammatory mediators [124]. Following viral infection, TLRs predominantly employ either of two pathways: the MyD88-dependent pathway or the TRIF-dependent pathway, via different TIR-domain containing adaptor proteins, such as MyD88, Mal (MyD88 adaptor-like protein), TIRAP (TIR-associated protein), TRIF and TRAM [125]. Recruitment of adaptor proteins brings about the activation of IRF-3/7 transcription factors, which culminates in the production of IFNs-α/β and chemokines including chemokine C-C motif ligands (CCLs) and C-X-C motif ligands (CXCLs) [126]. More and more evidence suggests the involvement of TLRs in multiple inflammation-driven diseases, along with pro- and anti-inflammatory cytokines, growth factors, and tumor suppressors [126]. Various TLR-based immune adjuvants have been validated for multiple clinical applications, including cancer immunotherapy [127].

**Figure 3:**
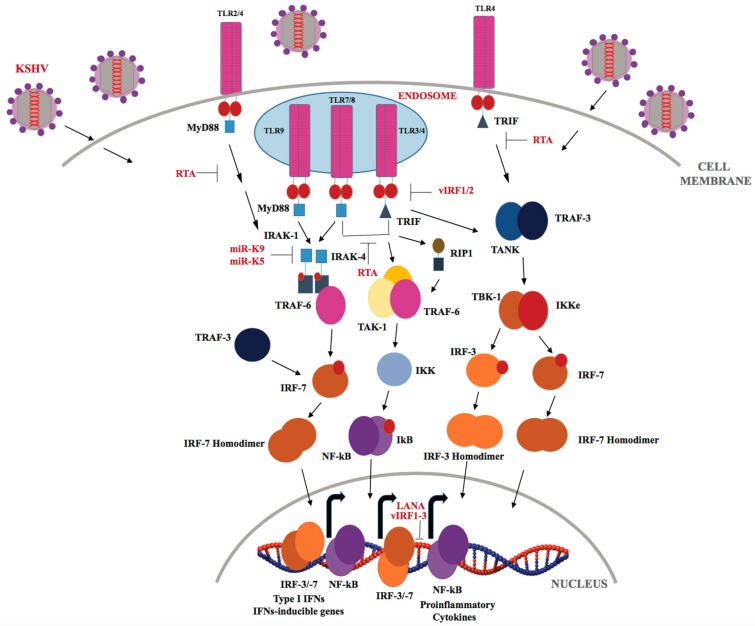
KSHV regulation of the TLR signaling pathway. Upon KSHV infection, TLRs that are expressed either on the cell surface (TLR-2 and 4) or in endosomes (TLR-3, 4, 7, 8, and 9) recruit TIR-domain containing MyD88 and TRIF adaptor molecules. The MyD88 and TRIF molecules then recruit downstream molecules, culminating in the activation of transcription factors such as IRF3/7 and NF-κB, which regulate the production of type I IFNs and inflammatory cytokines.

KSHV has been shown to be recognized by TLRs throughout its lifecycle (Figure 3). The Damania’s group showed that KSHV infection of human monocytes upregulates TLR-3, IFN-β, CCL2, and CXCL10 transcripts [128,129]. However, KSHV-encoded vIRFs (viral interferon regulatory factors-1/2/3) inhibit TLR-3-mediated activation of IFN-responsive promoters and suppress TLR-3 and CXCL10 gene expression [129]. KSHV encodes four viral IRFs (vIRFs 1-4), that show homology with cellular IRFs, and are negative regulators of cellular IFN immune responses [92,129]. KSHV vIRF-1 (K9) and vIRF-2 (K11/K11.1) repress IRF-3-mediated IFN-β production upon TLR-3 activation, though via different mechanisms [92]. According to a recent report by Jacobs et al. [130], vIRF-1 interacts with the cellular ISG15 E3 ligase HERC5 and decreases global ISGylation associated with TLR-3 activation. Previous studies have shown that vIRF-2 reduced the activation of the ISRE (IFN-induced interferon-response element) promoter through de-regulation of ISGF-3 (IFN-stimulated gene factor-3) [131]. KSHV infection of endothelial cells downregulates TLR-4 signaling by vGPCR-mediated ERK activation and vIRF-1, which, in turn, suppresses expression of TNF-α, IL-1β, IL-6 and IFN-β [132]. In THP-1 monocytes, KSHV infection results in inhibition of TLR-2/-4 signaling [133]. Furthermore, TLRs also play an important role during reactivation of KSHV from latency as TLR-7/-8 activation with a TLR-8 ligand, i.e., ss-polyuridine (a synthetic ssRNA homolog), was shown to trigger KSHV reactivation in latently infected B lymphocytes [134]. In plasmacytoid dendritic cells, TLR-9 has been reported to recognize KSHV, and lead to upregulation of CD83, CD86 and elevated IFN-α production [135].

The immediate-early lytic protein, RTA, which jump-starts the entire lytic replication cascade, has been shown to utilize several different strategies to inhibit TLR-mediated signaling, and consequently block inflammatory cytokines and type I IFN production. RTA has been found to regulate IFN production by targeting IRF-7, TRIF, TLR-2, and TLR-4 receptors [136]. RTA was also recently reported to down regulate MyD88 expression through the ubiquitin (Ub)-proteasomal degradation of MyD88, thereby blocking the TLR-4 signaling mediated IFN production and NF-κB activity [137]. KSHV-encoded miRNAs, miR-K12-9 and miR-K12-5 are found to regulate TLR/IL-1R signaling by suppressing IRAK1 and MyD88 protein expression [138]. As reported by Yang et al. IRAK1 kinase is constitutively phosphorylated and essential for PEL survival in culture and IRAK1-driven TLR signaling is a driving force for the PEL growth [139]. 

### 3.2. Intracellular NLRs, RLRs, and Cytosolic DNA Receptors in KSHV Infection 

NLRs are key sensors of intracellular pathogens in the host cell cytoplasm (Reviewed in [140,141]). The NLR family comprises of more than 23 cytoplasmic receptor proteins with a central nucleotide-binding domain (NOD), C-terminal leucine-rich repeats (LRRs), and an N-terminal protein binding motif, composed of a caspase-recruitment domain (CARD), or a pyrin domain (PYD), or a baculovirus inhibitor of apoptosis protein repeat (BIR) domain [115,141]. NLRs can activate the formation of large multi-scaffold protein complexes called inflammasomes, that are composed of an adaptor protein called ASC (apoptosis-associated speck-like protein containing a CARD) [142], pro-caspase-1, and an oligomer of a particular NLR. To date, several families of NLR inflammasomes have been identified, including NLRX1, NLRP1, NLRP3, and NLRC4. Among these, NLRP3 is the most studied and best characterized NLR family member [143]. Oligomerization of NLRs and their adaptor through CARD-CARD interactions is functionally important for regulation and activation of pro-inflammatory caspase 1, which results in the cleavage and maturation of pro-IL-1β and pro-IL-18 to their active forms IL-1β and IL-18, respectively [142,144]. Increased expression of proinflammatory mediators IL-1β and IL-18 has been observed to induce pyroptosis, defined as a caspase 1-mediated programmed cell death [145]. NOD1 and NOD2, the two predominant members of non-inflammasome NLRs are shown to activate IRF-3/7, NF-κB and MAPK signaling pathways in response to different PAMPs [146,147].

**Figure 4:**
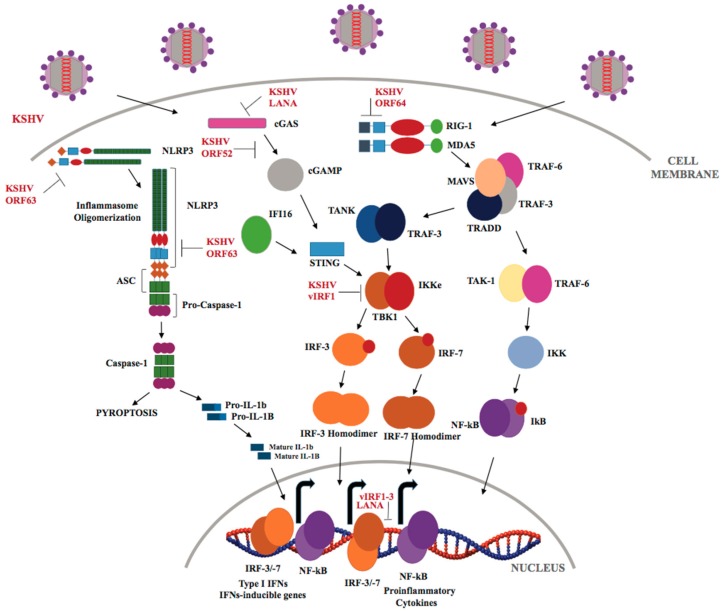
KSHV regulation of NLR, RLR, and cGAS-STING signaling pathways. Following KSHV entry into the host cell, the capsid extrudes KSHV dsDNA in the cytoplasm which is sensed by cGAS resulting in the STING/TBK1/IRF3 signaling and type I IFNs production. KSHV dsDNA is transcribed into dsRNA polymerase III and sensed by RIG-I and MDA-5, leading to activation and polymerization of MAVS and activation of NF-κB. KSHV also controls the inflammatory responses via NLRP-1/3-dependent caspase-1 activation and inhibition of IL-1β and IL-18 production.

KSHV ORF63, a viral homolog of cellular NLRP1, reduces caspase-1 activity and lowers the production of “alarm” pro-inflammatory cytokines (Figure 4), IL-1β and IL-18, by altering the interaction between NLRP1 and pro-caspase 1 through its association with NLRP1 oligomerization domains. Additionally, ORF63 inhibits NOD2 and NLRP3 inflammasome responses to suppress NLR-mediated signaling [148]. Recently, the ubiquitously expressed NLRX1, a negative regulator of the type I IFN response has been shown to play an important role in KSHV reactivation from latency by inhibiting MAVS-dependent IFN-I signaling [149]. 

RLRs are long recognized as intracellular viral dsRNA sensors in the cytoplasm (reviewed in [150]). However, there are reports of their involvement in the sensing of DNA viruses, expanding their repertoire as a cytoplasmic viral nucleic acid sensor [151]. The RLR family of proteins currently consists of three known members, namely RIG-I/DDX58, the melanoma differentiation-associated gene 5, MDA5/IFIH1, and the regulatory homologue laboratory of genetics protein 2, LGP2/DHX58 [152]. These RLR proteins are widely expressed in different cell types and are characterized by a central DExH/D-box RNA helicase domain and a C-terminal domain (CTD). In addition, RIG-I and MDA5 have tandem N-terminal CARD domains that activate signaling cascades to induce antiviral pro-inflammatory as well as type-I IFN-stimulated genes (ISGs). RIG-I primarily recognizes short dsRNA with an adjacent 5′- tri- or di- phosphate moiety, whereas MDA5 binds to internal sites in the long dsRNA with no end specificity [153]. In the absence of dsRNA, RIG-I is maintained in an inactive conformation where the CARD domains are bound by the RIG-I C-terminal repressor domains and cannot interact with the membrane-bound mitochondrial antiviral signaling adaptor protein, MAVS/IPS-I/VISA/CARDIF [154]. However, upon dsRNA binding, RIG-I undergoes conformational rearrangements in favor of CARD-CARD interactions between RIG-I and MAVS, [155,156] leading to activation of kinases that activate NF-kB and IRF-3/7 and in turn, induce the promoter for type I IFNs (IFN-α/β) [10,157,158]. In contrast to RIG-I, dsRNA binding triggers the helicase domains of MDA5 to wrap around dsRNA leading to a 20° rotation of MDA5 CTD. The rotation of MDA5-CTD promotes ATP-dependent MDA5 cooperative filament formation on dsRNA and induces oligomerization of MDA5 CARDs, which in turn, forms a scaffold for oligomerization of MAVS CARD [153,159,160]. LGP2, that lacks CARD domains for signaling, is thought to influence IFNs production by regulating the activity of RIG-I and MDA5. Though the exact function of LGP2 in viral infections is still unclear, it is proposed that LGP2 inhibits IFN induction by sequestering PAMPs from RIG-I [161]. A study by Childs et al. reported that LGP2 plays an important role in sensitizing MDA5 to activation by dsRNA [162].

In comparison to TLR and NLR signaling, little is known about the role of RLR signaling during KSHV infection. West et al. showed that both RIG-I and MAVS play a role in IFN-β production following KSHV infection and can suppress KSHV replication and/or reactivation. Nevertheless, KSHV-encoded deubiquitinase (DUB), ORF64, has been shown to inhibit RIG-I signaling and activation of IFN-β via the prevention of ubiquitination of RIG-I [163]. Hence, RIG-I or MDA5 could be further exploited as promising oncogenic targets.

Cytosolic DNA sensors belong to the most recently identified members of the PRR family, and hence, lack full characterization among known PRRs. To date, several intracellular DNA sensors have been identified. DNA-dependent activator of IFN-regulatory factors (DAI), absent in melanoma 2 (AIM2), gamma interferon-inducible protein 16 (IFI16), and cyclic guanosine monophosphate-adenosine monophosphate (cyclic GMP-AMP synthase; cGAS) are the major cytosolic DNA sensors involved in innate immune responses, following viral infection [164,165,166,167,168,169]. However, unlike, TLR and NLR signaling, not much is known about the viral sensing by the cGAS-STING pathway. Upon binding of cytosolic DNA, cGAS dimerizes and generates cyclic-GMP-AMP (cGAMP), which binds to a deep pocket in the adaptor protein STING/MITA/MPYS/ERIS located in the ER membrane [170]. STING acts as a scaffold to recruit IFN-inducing proteins, namely TBK-1 (TNFR-associated NF-kB kinase (TANK)-binding kinase-1) and transcription factor IRF-3, leading to production and secretion of IFNs [171,172]. IFI16, a recently characterized receptor for dual sensing of nuclear and cytosolic viral DNA, has been identified to play a key role in the activation of STING/TBK1/IRF-3 signaling, required for induction of several IFNs, ISGs, and inflammasomes [173,174].

IFI16, a cytosolic DNA receptor, has been recognized as a stimulator of innate immunity upon KSHV infection. KSHV DNA has been shown to activate IFI16, resulting in the formation of IFI16-ASC-procaspase-1-inflammasome assembly to activate caspase-1 and IL-1β (Figure 4) [175,176]. The major products of inflammasome activation, IL-1β and IL-18, are pro-tumorigenic and shown to play an important role in tumor-mediated angiogenesis, hence blocking their function may suppress tumor progression. A recent study identified BRCA1 as an important constituent of IFI16-ASC-procaspase-1-inflammasome assembly that promotes induction of inflammasome and IFN-β responses during de novo KSHV infection [177]. Roy et al., for the first time, has recently demonstrated the role of IFI16 in maintenance of KSHV latency in KSHV-infected PEL cell lines, by functioning as a transcriptional repressor for viral lytic promoters [178]. Cytoplasmic isoforms of KSHV LANA recruit and antagonize the function of cGAS to inhibit cGAS-STING mediated restriction of KSHV lytic replication and promote reactivation from latency [179]. KSHV ORF52, a gammaherpesvirus specific virion tegument protein, disrupts the cGAS signaling pathway by inhibiting the enzymatic activity of cGAS and, as a consequence, cGAMP synthesis [180]. KSHV vIRF1 has been reported to block cGAS-STING-mediated antiviral immunity in endothelial cells by de-stabilizing TBK1 binding to STING [181]. Since intracellular DNA receptors play diverse yet critical roles during de novo infection, as well as in KSHV reactivation from latency, KSHV-encoded cGAS/STING/TBK-1 inhibitors may provide targets for developing diagnostic approaches for KS-mediated tumorigenesis.

## 4. Conclusions and Future Perspectives

It has been well established that viral immune evasion strategies play a critical role in virus-induced tumor escape from the host’s surveillance machinery. Accumulating evidence has indicated the involvement of nearly all known PPRs in diseases associated with each of the seven oncogenic viruses. Activation of PRR-mediated signaling, through interaction between PRRs and viral PAMPs at the surface of the cell, in the endocytic compartments, in the cytoplasm, or in the nucleus, promotes a diverse range of inflammatory, innate and adaptive immune responses. Hence targeting these cellular immune sensors, immunomediators and associated pathways represents a promising cancer prevention/treatment approach. 

Interestingly, prolonged and dysregulated activation of PRRs reroutes the protective PRR-triggered pathways, key transcription factors and specialized cells of the immune system, causing chronic inflammation that facilitates tumor progression [182]. It has been proven that abnormal expression of PRRs favors tumorigenesis via release of various proliferative, anti-apoptotic, and angiogenic factors that contribute to the tumor microenvironment [183]. Mounting evidence inextricably suggests the association of nearly all known members of PRR families with increased tumor incidence and disease severity in several human malignancies (Table 2) [184,185]. Several tumor models and hematological malignancies display aberrant expression of TLRs, in particular TLR-4 and TLR-9 [186,187]. For instance, the upregulation of TLR-4, expressed on head and neck squamous cell carcinoma, has been found to favor tumor growth and development [188]. Involvement of TLR-4/MyD88 signaling in epithelial ovarian cells is shown to promote paclitaxel chemoresistance as well as tumor progression [189]. TLR activation, when combined with inflammatory responses, causes inflammation-driven tumor progression. Elevated expression of major inflammasome activation products, IL-1β and IL-18, have been implicated in gastrointestinal and colitis-associated cancer [190].

The vast majority of studies are providing mounting evidence for the PRR involvement in KSHV recognition (via KSHV-PAMPs) and KS carcinogenesis [114]. Similar to other oncoviruses, KSHV has achieved a balance with its host to limit its recognition and ensure its long-term survival following infection. However, the exact knowledge of these intricate molecular mechanisms and the full spectrum of KSHV-encoded proteins that antagonize PRRs remains incompletely understood. Further studies to shed light into the important structure-function analyses of viral PAMP-host PRR interaction and context-specific relevance of KSHV-triggered PRRs modulation during KS tumorigenesis may stimulate the development of novel prophylactic and therapeutic approaches against KSHV-positive malignancies, and may contribute to long term clinical benefits. 

## Figures and Tables

**Table 1:** Summary of current biomarkers in acquired immune deficiency syndrome (AIDS)-associated KS.

Cellular Markers	Viral Markers/Proteins	Cellular Oncogenes	Growth Factors/Inflammatory Cytokines
CD4 Count [41,61,62]	KSHV Virus [63,64,65]	bcl-2 [49,66,67]	CD31 [57,64,68,69]
	LANA/ORF73 [64,65,66,70]	c-kit [71,72]	CD34 [57,68,69]
	vCyclin/ORF72 [67,73,74]	p53 [46,75,76]	CD36 [51,77,78]
	vFLIP/ORF71 [49,74,79,80]	pRb [47,81,82]	CD138/Syndecan-1 [83,84]
	K12/Kaposins [49,85,86]		FLI1 [57,87,88]
	miRNAs [48,85,86,89]		D2-40 [57,90,91]
	vIRFs [48,92,93,94]		Podoplanin [54,55,95]
	vIL-6 [49,50,59]		VEGFR3 [39,56,95,96]
	vGPCR/ORF74 [48,97]		LYVE-1 [39,54,95,96]
			TNF-α [58,59]
			bFGF [39,58,98,99]
			Oncostatin M [58,60,100]
			vIL-6 [49,50,59]
			IL-I [58,59,101]

**Table 2:** Summary of key PRRs and associated human cancers.

PRRs	Cellular Localization	PAMPs	Associated Tumors
TLR-1/2	Plasma membrane	Lipoprotein	Colon cancer [191]
TLR-3	Endosome	Double stranded (ds)RNA	Melanoma, Colorectal adenoma, low-grade B-cell Lymphoma, Solid tumors [192,193]
TLR-4	Plasma membrane	Lipopolysaccharides	Lung cancer, Non-Hodgkin’s lymphoma [194]
TLR-5	Plasma membrane	Flagellin	Advanced/Metastatic solid tumors [195]
TLR-7/8	Endosome	Single stranded (ss)RNA	Ovarian cancer, Solid tumors [196,197,198]
TLR-9	Endosome	CpG-DNA	Colorectal cancer, Breast cancer, Chronic Lymphocytic Leukemia [199,200,201]
MDA5	Cytoplasm	Long dsRNA	Solid tumors [202]
STING	Cytoplasm	dsDNA	Advanced/Metastatic solid tumors [203]
